# A New Method of Inviting Sleep without Drugs

**Published:** 1900-07

**Authors:** J. B. Learned

**Affiliations:** Northampton, Mass.


### A NEW METHOD OF INVITING SLEEP WITHOUT DRUGS.[1]

BY DR. J. B. LEARNED, NORTHAMPTON, MASS.

Mr. Chairman and Gentlemen,—I am conscious of the honor as well as the responsibility of appearing before you on this occasion. Your representative has asked me to tell you how to go to sleep.

In 1880 a severe injury to the brain and nervous system came to me, and for many years following it seemed to be necessary for me to learn how to go to sleep. I had become convinced that the effects of hypnotic drug-taking were more damaging than sleeplessness left to itself. After many years' experimenting, I became convinced that we could command the conditions, so to speak, in bed as readily as can the soldier and the huntsman prepare an appetite for a dinner. When I used the muscles sufficiently through the day, sleep came readily on retiring; but this was not always the case, and on going to bed I found I was not sufficiently prepared by being "all-round" tired. When I needed muscular exercise, and

---

[1] Read before The New York Institute of Stomatology, March 6, 1900.

went to the basement and ran a wood-saw long enough, I could return and readily go to sleep. Gymnastics in the bath-room, with hot and cold water adjuncts, served me a good purpose also. A walk of two or three miles around the square and back had the same effect. I felt tired then, and was ready for sleep.

This mode of preparation, however, has some unattractive features. Dressing and undressing in the middle of the night is one of them. It occurred to me here, under these circumstances, that muscular effort might be employed while in my ordinary recumbent position in the bed. I had found sometimes that the tired sense experienced while engaged in wood-sawing or walking disappeared when I was again in my bed, and the sleepless condition reappeared.

What are the obstructions in the way of sleep when we fail to reach it promptly on retiring? I have no time to talk upon remote causes. Civilization, tea, coffee, tobacco, the modern cook-book, and the drug habit, all potent factors in preparing the foundations for insomnia, I omit. I am to deal with the one obstacle which the brain-worker has to meet immediately on retiring, or at three or four o'clock in the morning,—viz., automatic thinking. Without attempting to quote authority, I will say the brain is a great workshop, with machines, shafts, pulleys, and belts. The work of the brain is thinking. The power is fresh arterial blood. The immediate obstacle to sleep seems to be automatic thinking. Power being furnished, some belt is on, and one of the many nerve-centres persists in operating after work-hours. Now, since the machine always stops when the power is shut off securely, if we turn off the belt the thinking would stop and sleep come. Without going into the details of giving you the thousand and one contractions and relaxations I have made use of for the purpose of drawing the blood from the head, let me outline a few of the simple motionless and invisible exercises that serve generally a very good purpose. Let us take the respiration in hand.

Ordinarily the number of inspirations and expirations are twelve or fifteen. Reduce this number to four or six, making each inspiration full, regular, deep, prolonged; and let the expirations be carried on in the same manner. Thus the will-power is employed, and the muscles and respirations are called upon to an unusual extent. A part of the power that was used in the brain to carry on automatic thinking has now been turned into new channels. Keep this new mode of respiration under control, after the manner just described, for a few minutes, and the chances are more than equal that an equilibrium has already been established in the circulation, and that the automatic process in the brain has been checked. As soon as this is done sleep comes.

Take another exercise: Immediately upon going to your bed measure your length by reaching for the footboard and the headboard at the same time. In doing this you are exercising a large number of the trunk muscles and the muscles of the extremities. Hold this position for a length of time, until a sense of fatigue comes to the muscles engaged, and sleep may follow. Both these exercises may be carried on at the same time,—controlling the respirations and elongating the body to its utmost by trying to touch the headboard with the head and the footboard with the feet.

Here is another exercise: Remove the pillow for the time being. Lift the head a half-inch from its resting-place; hold it there, lying upon the back, until the muscles become weary; it will take but a very short time. Drop the head now, retaining the same position in bed, and raise the right foot a half-inch from its resting-place. You have now to hold the weight of the clothes and weight of the foot. The sense of fatigue soon comes to these muscles. Drop this foot and elevate the other in like manner. Hold for the same length of time, until the sense of fatigue is here manifested. Return the foot to its place. If you keep the regularity of respirations during these exercises of head and foot elevation under the constant watchfulness of the will, you have drawn upon the reservoir of power by calling blood to other muscles used, and you have relieved the parts of the brain having an over-supply, which caused the automatic activity.

Still another group of muscles may be brought into use by turning upon the side, lifting the head by the use now of a new set of muscles, hold until, as before, your sensation says "enough," then let the head go down. Remaining on the same side lift the limb enough to sense the weight of the clothes as before, and hold until the call comes to go down, which you will obey without prolonged exertion. Turn now onto the opposite side, lift the head as before, use the opposite set of muscles, remaining for the same length of time to change as before the limb that is now uppermost. Hold still again the limb with its covering as before, until fatigue warns you to drop it.

Still another exercise, which may be engaged in with the same quiet and almost invisible results: Extend your arms by your sides, as though you would reach for the same footboard as before; contract every muscle in the arms; hold as before until the sense of fatigue warns you to change. Every muscle that has been used in these exercises has required the same power that has been in use without our consent or permission to carry on the automatic thinking of the brain. Bringing into use thus the muscles of all parts of the body, we have called power away from the over-supplied brain. Our method might be termed that of counterirritation. These exercises act as mustard-plasters to draw blood to one part and relieve over-distention in another part.

Now, it must be borne in mind that this mode of walking round the square, sawing wood with a dull saw, taking hot and cold baths, going through with a variety of gymnastic exercises at the open window, has been duplicated by the exercises we have gone through with in bed without dressing or undressing. Let it be borne in mind also that the one aim has been to take away power from that part of the brain whose juvenile pranks have disturbed us for so many nights and to transfer it to other parts. In other words, we have been turning off belts from one shaft and turning on belts to other shafts in this great machine-shop.

If the exercises be moderate that I have outlined, no addition has been made to the heart-beats. If persisted in with much exertion, the heart-beat will be accelerated as it would be by running instead of walking; as it would be by sawing a half-cord of wood in an hour instead of in two hours. Each individual is a law to himself here as elsewhere. The exercises which will fit one case do not so readily fit another, and the amount of exertion which one person can make and endure might be altogether too much for another person. So the very moderate efforts which I have outlined, always stopping at the first sensation of fatigue, might be entirely insufficient for some people. The robust, stalwart, vigorous brain-worker, who has allowed his muscles during the day to lie dormant, may well undergo more severe trial than the more slender, more nervous, and more broken man of mature years. Here let me say the condition of the heart, the condition of the lungs, the condition of the nerve-centres, of the digestive organs, and of the general nutrition are all factors in determining just what is best for each individual, not only in his day's work, but in the extemporized exercises which I have just outlined to you. Do not overdo it. Heart failure appears as the cause of sudden exit in altogether too many cases.

I hope no one of you will furnish a reporter, to-morrow morning, the opportunity to say that heart-failure was the cause of his departure; and that the cause was the trial of the new method of inviting sleep without drugs. You can obviate this. Let your family physician listen to your heart; examine the molecular condition of your nerve-centres and the normal capacity of your lung-power.

